# Expression Analysis of Mesenchymal Stem/Progenitor Cell Markers on Chondrocytes in Ossification of the Posterior Longitudinal Ligament

**DOI:** 10.7759/cureus.90749

**Published:** 2025-08-22

**Authors:** Takashi Tsuji, Masashi Nakatani, Kaori Tajima, Shingo Maeda, Ichiro Kawamura, Nobuyuki Fujita, Harumoto Yamada

**Affiliations:** 1 Orthopedic Surgery, Fujita Health University, Aichi, JPN; 2 Orthopedic Surgery, National Hospital Organization Tokyo Medical Center, Tokyo, JPN; 3 Rehabilitation and Care, Seijoh University, Aichi, JPN; 4 Bone and Joint Medicine, Kagoshima University Graduate School of Medicine and Dental Sciences, Kagoshima, JPN; 5 Orthopedic Surgery, Kagoshima University Graduate School of Medicine and Dental Sciences, Kagoshima, JPN

**Keywords:** cell surface marker, chondrocyte, heterotopic ossification, mesenchymal stem/progenitor cell, posterior longitudinal ligament

## Abstract

Introduction: One hypothesis of ossification of the posterior longitudinal ligament (OPLL) pathogenesis is that pluripotent mesenchymal stem/progenitor cells (MSCs) differentiate into chondrocytes and that heterotopic ossification occurs via endochondral ossification. However, studies on the origin and characteristics of these ectopically appearing chondrocytes are limited. The purpose of this study was to investigate the characteristics of chondrocytes in human OPLL tissue, with a particular focus on MSC markers.

Methods: OPLL samples were collected during surgery from four patients with cervical or lumbar OPLL. We investigated the expression of cell surface markers of MSC by reverse transcription-polymerase chain reaction (RT-PCR) and immunostaining.

Results: RT-PCR analysis revealed the expression of CD73, CD90, CD105, and platelet-derived growth factor receptor α (PDGFRα) in OPLL tissue. Immunostaining analysis also demonstrated that the chondrocytes in ossified tissue co-expressed CD73, CD90, CD105, and PDGFRα.

Conclusions: Chondrocytes in the interstitium of the ossified tissue co-expressed MSC markers CD73, CD90, CD105, and PDGFRα, suggesting that ectopically appearing chondrocytes were derived from MSCs. These results indicated that MSCs are deeply involved in the pathogenesis of endochondral ossification in OPLL.

## Introduction

Ossification of the posterior longitudinal ligament (OPLL) is a disease in which the spinal ligament is ectopically ossified. This disease is thought to be more common in Asian populations than in Caucasian populations; the prevalence of spinal ligament ossification is 6.3% in cervical OPLL, 1.6% in thoracic OPLL, and 0.3% in lumbar OPLL in the Japanese population [1]. The underlying various etiologies, such as genes [2], dynamic factors [3], inflammation [4], metabolic abnormalities [5], metabolites [6], and dietary habits [7,8] have been reported as the cause of OPLL; however, the mechanism of ossification is still unknown, thus there is no definitive treatment.

Mesenchymal stem/progenitor cells (MSCs) are a heterogeneous population of multipotent stem cells with self-renewing capacity and the ability to differentiate into bone, adipose tissue, and cartilage. These cells were first discovered in bone marrow by Friedenstein et al. and were initially called colony-forming unit-fibroblasts [9]. Recently, MSCs have been widely used in the treatment of various inflammatory and degenerative diseases and cancers due to their ability to repair damaged tissue, differentiate into different cell types, and secrete a variety of soluble mediators with pleiotropic effects [10,11].

One hypothesis is that multipotent MSCs differentiate into chondrocytes and ossification occurs via endochondral ossification [12,13]. However, studies on the origin and characteristics of these ectopically appearing chondrocytes are limited. Previous reports have shown that S100 protein-positive chondrocytes co-express MSC markers using the ossification of the ligamentum flavum (OLF) samples [13], but reports examining chondrocyte characteristics using OPLL samples are limited [14,15], and no reports exist examining whether chondrocytes in the OPLL express MSC markers using multiple cell surface markers.

The objective of this study was to investigate the characteristics of chondrocytes in human OPLL tissue, with a particular focus on the presence or absence of MSC markers on the cell surface, employing molecular biological and histological methods.

## Materials and methods

The authors were members of the Study Group of the Investigation Committee on Ossification of the Spinal Ligaments in Japan, and this multicenter study was conducted in collaboration with the aforementioned study group between 2016 and 2021. The inclusion criteria were patients with cervical or lumbar OPLL diagnosed by computed tomography and consecutive patients who underwent OPLL resection. The exclusion criteria were non-operative patients, patients who did not consent to the study, and patients who underwent posterior decompression only without OPLL resection. Ethical approval was obtained from the institutional review board (IRB no. HM16-282). Written informed consent was obtained from all individual participants included in the study.

Human OPLL samples

Ossified tissue of the posterior longitudinal ligament was harvested from four consecutive cases of anterior surgery in patients with cervical or lumbar OPLL (Table 1). One sample was collected at Fujita Health University Hospital, and three samples were collected at Kagoshima University Hospital. The resected ossified ligament and surrounding tissue were preserved in ribonucleic acid (RNA) extraction liquid or formalin immediately after resection.

**Table 1:** List of OPLL samples RT-PCR: reverse transcription-polymerase chain reaction; HE: hematoxylin-eosin; IF: immunofluorescent; OPLL: ossification of the posterior longitudinal ligament

Case	Age	Gender	Ossified type / Level	Evaluation methods
1	70	Female	Mixed / C4-5	RT-PCR
2	33	Female	Circumscribed / L4-5	HE and IF staining
3	48	Male	Segmental / C5	HE and IF staining
4	59	Male	Circumscribed / L4-5	HE and IF staining

RNA extraction and RT-PCR

Total RNA was extracted using ISOGEN 2 (Nippon Gene, Tokyo, Japan). Around 500 ng/10 µl RNA were reverse transcribed into complementary deoxyribonucleic acid (cDNA) using a PrimerScript RT master mix (Takara Bio, Kusatsu, Japan). The polymerase chain reaction (PCR) was performed with EX Taq (Takara Bio, Kusatsu, Japan) under the following cycling conditions: 95°C for three minutes, followed by 35 cycles of amplification (94°C for five seconds, 60°C for 20 seconds, 72°C for 10 seconds), and final incubation at 72°C for five minutes. The expressions of clusters of differentiation 73 (CD73), CD90, CD105, and platelet-derived growth factor receptor α (PDGFRα, CD140a) were evaluated. Specific primer sequences used in this study are shown in Table 2.

**Table 2:** Primer sequences PDGFRα: platelet-derived growth factor receptor α; GAPDH: glyceraldehyde-3-phosphate dehydrogenase

Gene	Forward	Reverse
CD73	5' CCAGTACCAGGGCACTATCTG 3'	5' TGGCTCGATCAGTCCTTCCA 3'
CD90	5' ATCGCTCTCCTGCTAACAGTC 3'	5' CTCGTACTGGATGGGTGAACT 3'
CD105	5' TGCACTTGGCCTACAATTCCA 3'	5' AGCTGCCCACTCAAGGATCT 3'
PDGFRα	5' TCCTCTGCCTGACATTGACC 3'	5' TGAAGGTGGAACTGCTGGAAC 3'
GAPDH	5' ACCCACTCCTCCACCTTTGA 3'	5' TTGCTGTAGCCAAATTCGTTG 3'

Histological examination and immunostaining

Hematoxylin-eosin (HE) and immunofluorescent (IF) staining were performed using three human OPLL samples (Table 1). Surgically resected human OPLL samples were fixed with formaldehyde neutral buffer solution and then embedded in paraffin.

For IF staining, paraffin-embedded sections were treated with Target Retrieval Solution, pH 9.0 (DAKO, Glostrup, Denmark) for 10 minutes. Around 0.5% Triton-X 100 was used for membrane permeabilization for five minutes. Sections were blocked with Protein-Block Serum-Free reagent (DAKO, Glostrup, Denmark) for 10 minutes and incubated with primary antibodies for one hour at room temperature. CD73 (1:200, Cell Signaling Technology, MA, USA), CD90 (1:200, Cell Signaling Technology, MA, USA), CD105 (1:200, Cell Signaling Technology, MA, USA), and PDGFRα (1:100, R&D Systems, MN, USA) were used for primary antibodies. Alexa Fluor 488 and 594 (Thermo Fisher Scientific, MA, USA) were used for secondary antibodies. Antifade mounting medium was used to prevent rapid photobleaching. To examine the distribution of PDGFRα-positive cells, 3, 3’Diaminobenzidine (DAB) staining was used.

Stained sections were photographed using a fluorescence microscope BX51 equipped with a DP71 camera (Olympus, Tokyo, Japan).

## Results

RT-PCR

**Figure 1:**
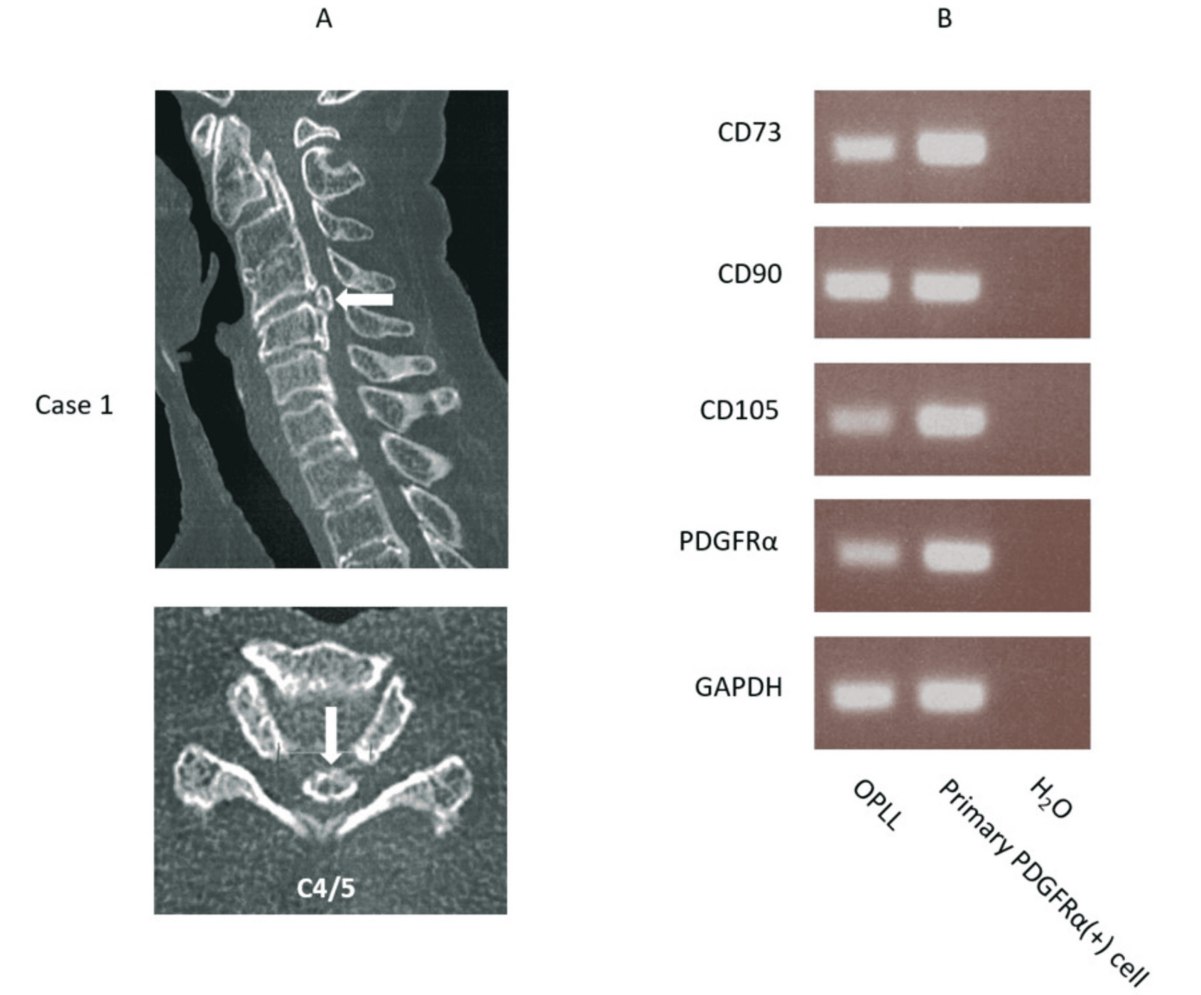
Expressions of CD73, CD90, CD105, and PDGFRα mRNAs A: cervical C4/5 ossification of the posterior longitudinal ligament (OPLL) tissue from a 70-year-old female is subjected to reverse transcription-polymerase chain reaction (RT-PCR) (white arrows indicate OPLL sampling sites); B: expressions of CD73, CD90, CD105, and platelet-derived growth factor receptor α (PDGFRα) mRNAs are detected in OPLL tissue. Primary human PDGFRα-positive cells are used as a positive control. H₂O is used as a negative control. Each RT-PCR is conducted using 500 ng/10 µl RNA.

The first case of cervical OPLL tissue was analyzed for the presence or absence of cell surface markers using reverse transcription-polymerase chain reaction (RT-PCR) (Figure 1A). Primary PDGFRα-positive cells that were isolated from human muscle were used as a positive control. The results revealed that expression of CD73, CD90, CD105, and PDGFRα, which were the markers for MSCs, was confirmed in OPLL tissue (Figure 1B).

Histological examination and immunostaining

**Figure 2:**
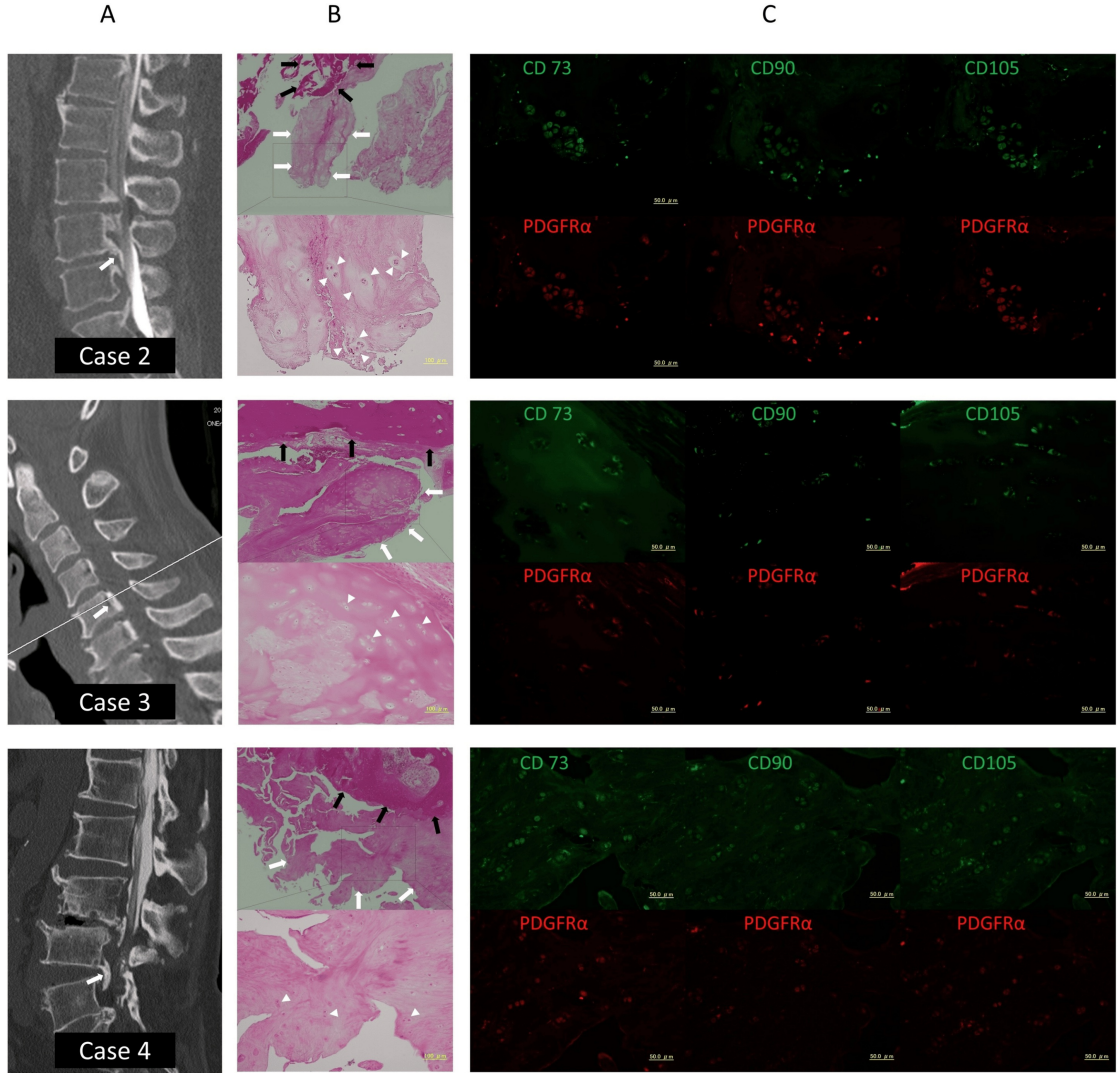
Hematoxylin-eosin staining and mesenchymal stem/progenitor markers expression Hematoxylin-eosin (HE) staining and immunofluorescent (IF) staining of lumbar or cervical ossification of the posterior longitudinal ligament (OPLL). A: computed tomography images of patients (white arrows indicate OPLL sampling sites); B: HE staining (black arrows indicate mature bone, white arrows indicate interstitial spaces, and white triangles show chondrocyte clusters and surrounding cartilage matrix); C: IF staining shows that CD73, CD90, and CD105 (green)-positive chondrocytes co-express platelet-derived growth factor receptor α (PDGFRα) (red).

Next, cervical and lumbar OPLL tissues were used for histological examination to confirm the expression of MSC markers at protein levels and whether or not they were expressed in chondrocytes (Figure 2A). HE staining showed mature bone and interstitial spaces in OPLL tissue. The interstitial spaces contained several clusters of chondrocytes surrounded by cartilage matrix in the neighborhood of mature bone. Pale red collagen fibers stained with eosin were observed around the cartilage matrix, a finding consistent with fibrocartilage tissue (Figure 2B).

Immunofluorescent staining showed that CD73, CD90, and CD105 were positive on chondrocytes within fibrocartilage. These CD73, CD90, and CD105 positive chondrocytes co-expressed PDGFRα. On the other hand, these markers were not expressed in mature bone (Figure 2C).

**Figure 3:**
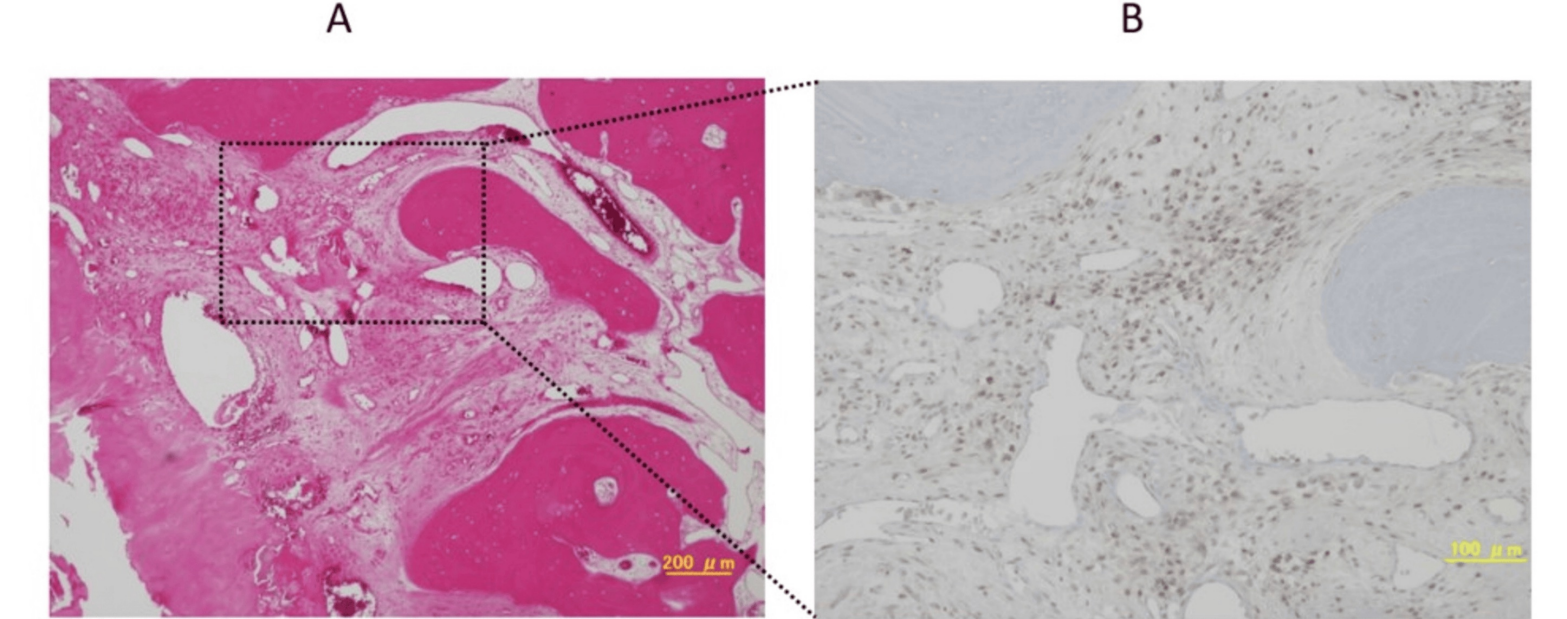
Hematoxylin-eosin staining and 3,3'Diaminobenzidine staining of PDGFRα A: HE staining; B: platelet-derived growth factor receptor α (PDGFRα)-positive cells are widely distributed in non-ossified interstitial space, and PDGFRα is positive in a variety of cell morphologies.

PDGFRα-positive cells were widely distributed in the non-ossified interstitium. PDGFRα was positive not only in chondrocytes but also in various cell types (Figure 3).

## Discussion

This is the first study to show that chondrocytes in human OPLL tissue expressed multiple MSC markers such as CD73, CD90, CD105, and PDGFRα. Due to limited opportunities to obtain ossified posterior longitudinal ligaments from patients, previous studies have often used the ligamentum flavum instead of the posterior longitudinal ligament [12,13], but this study evaluated the posterior longitudinal ligament tissue itself.

MSCs are multipotent progenitor cells that possess the ability to undergo in vitro self-renewal and differentiate into various mesenchymal lineages. Advances in stem cell technology offer new possibilities for patients with diseases and disorders. Stem cell-based therapy, which includes multipotent MSCs, has recently become important in regenerative therapies, such as tissue regeneration, immunological modulation, anti-inflammatory qualities, and wound healing [10].

Heterotopic ossification is the abnormal bone formation in musculoskeletal tissues that results from inappropriate differentiation of MSCs. There are some reports that multipotent MSCs play important roles in the pathological development of several heterotopic ossifications, such as heterotopic ossification around the hip joint [16] and vascular calcification [17]. Although previous studies have also demonstrated that MSCs are involved in endochondral ossification of OLF [12,13], reports on the origin or cell surface markers of chondrocytes involved in endochondral ossification of OPLL are limited.

Although there is no consensus regarding the surface marker of human MSCs, the International Society for Cellular Therapy proposes minimal criteria as "MSC must express CD105, CD73, and CD90 and lack expression of CD45, CD34, CD14 or CD11b, CD79alpha or CD19, and HLA-DR" [18]. Thus, in this study, we selected CD73, CD90, and CD105 as the markers of multipotency.

PDGFRα is one of the two PDGFR subunits (α and β), which form homo- and heterodimers. This receptor binds to certain isoforms of PDGF and thereby becomes active in cell signaling that elicits responses such as cellular proliferation and differentiation. PDGFRα has been used as a specific isolation marker for MSCs in human skeletal muscle [19], and recent studies indicated that PDGFRα-positive stem/progenitor cells have an osteogenic identity in fields such as bone formation and fracture repair [20], vascular calcification [17], and dental ossification [21] and are a major cell source of heterotopic ossification in the limb. Oishi et al. reported that human skeletal muscle-derived PDGFRα-positive progenitor cells have osteogenic potential and that PDGFRα-positive cells are the major source of ectopic ossification in soft tissue [16]. Using a mouse model of heterotopic bone formation, Agarwal et al. also reported that PDGFRα-positive mesenchymal cells aggregate in developing heterotopic ossification lesions and co-express SRY-box containing gene 9 (Sox-9), thereby resulting in endochondral ossification [22]. Furthermore, Bartoletti et al. reported that PDGFRα activation enhances Sox9 and Col2a1 expression and regulates MSCs differentiation towards chondrocyte progenitors [23]. Thus, in the present study, we selected PDGFRα as a supplemental marker for MSCs in addition to CD73, CD90, and CD105.

On the other hand, this study revealed that PDGFRα was expressed not only in chondrocytes but also in various types of cells in the interstitium of ossifying tissues. PDGFRα signaling has been reported to promote not only osteogenic and chondrogenic differentiation of MSCs, but also adipocyte and fibroblast differentiation [19,24,25]. Therefore, PDGFRα signaling could have a diverse role, and PDGFRα alone would not be a useful specific marker of chondrocyte progenitor cells in OPLL.

This study has several limitations, including the small sample size, the limited number of MSC markers examined, and the fact that MSC marker detection was limited to two methods: RT-PCR and immunostaining. In addition, there is no direct evidence that chondrocytes expressing CD73, CD90, CD105, and PDGFRα are involved in heterotopic ossification. Therefore, further studies are needed to determine the precise roles of chondrocytes that appear in OPLL tissue.

## Conclusions

In this study, we confirmed the expression of CD73, 90, 105, and PDGFRα, which are markers of MSCs, in human OPLL tissue by RT-PCR and clarified that these cell surface markers were co-expressed in chondrocytes by immunostaining. These findings indicated that the ectopically appearing chondrocytes were cells differentiated from MSCs, and furthermore, that these cell surface markers continued to be expressed even after differentiation from MSCs to chondrocytes. On the other hand, PDGFRα was expressed in various types of cells and was not a marker specific to chondrocytes. Characterization of ectopically appearing chondrocytes is expected to provide new insights into understanding the pathogenesis of OPLL.

## References

[1] Fujimori T, Watabe T, Iwamoto Y, Hamada S, Iwasaki M, Oda T (2016). Prevalence, concomitance, and distribution of ossification of the spinal ligaments: results of whole spine CT scans in 1500 Japanese patients. Spine (Phila Pa 1976).

[2] Nakajima M, Takahashi A, Tsuji T (2014). A genome-wide association study identifies susceptibility loci for ossification of the posterior longitudinal ligament of the spine. Nat Genet.

[3] Tanno M, Furukawa KI, Ueyama K, Harata S, Motomura S (2003). Uniaxial cyclic stretch induces osteogenic differentiation and synthesis of bone morphogenetic proteins of spinal ligament cells derived from patients with ossification of the posterior longitudinal ligaments. Bone.

[4] Kawaguchi Y, Nakano M, Yasuda T (2017). Serum biomarkers in patients with ossification of the posterior longitudinal ligament (OPLL): inflammation in OPLL. PLoS One.

[5] Ikeda Y, Nakajima A, Aiba A, Koda M, Okawa A, Takahashi K, Yamazaki M (2011). Association between serum leptin and bone metabolic markers, and the development of heterotopic ossification of the spinal ligament in female patients with ossification of the posterior longitudinal ligament. Eur Spine J.

[6] Tsuji T, Matsumoto M, Nakamura M (2018). Metabolite profiling of plasma in patients with ossification of the posterior longitudinal ligament. J Orthop Sci.

[7] Wang PN, Chen SS, Liu HC, Fuh JL, Kuo BI, Wang SJ (1999). Ossification of the posterior longitudinal ligament of the spine. A case-control risk factor study. Spine (Phila Pa 1976).

[8] Okamoto K, Kobashi G, Washio M (2004). Dietary habits and risk of ossification of the posterior longitudinal ligaments of the spine (OPLL); findings from a case-control study in Japan. J Bone Miner Metab.

[9] Friedenstein AJ, Chailakhjan RK, Lalykina KS (1970). The development of fibroblast colonies in monolayer cultures of guinea-pig bone marrow and spleen cells. Cell Tissue Kinet.

[10] Zhidu S, Ying T, Rui J, Chao Z (2024). Translational potential of mesenchymal stem cells in regenerative therapies for human diseases: challenges and opportunities. Stem Cell Res Ther.

[11] Taeb S, Rostamzadeh D, Mafi S (2024). Update on mesenchymal stem cells: a crucial player in cancer immunotherapy. Curr Mol Med.

[12] Asari T, Furukawa K, Tanaka S (2012). Mesenchymal stem cell isolation and characterization from human spinal ligaments. Biochem Biophys Res Commun.

[13] Chin S, Furukawa K, Ono A (2013). Immunohistochemical localization of mesenchymal stem cells in ossified human spinal ligaments. Biochem Biophys Res Commun.

[14] Sugita D, Yayama T, Uchida K (2013). Indian hedgehog signaling promotes chondrocyte differentiation in enchondral ossification in human cervical ossification of the posterior longitudinal ligament. Spine (Phila Pa 1976).

[15] Nakajima H, Watanabe S, Honjoh K, Okawa A, Matsumoto M, Matsumine A (2020). Expression analysis of susceptibility genes for ossification of the posterior longitudinal ligament of the cervical spine in human OPLL-related tissues and a spinal hyperostotic mouse (ttw/ttw). Spine (Phila Pa 1976).

[16] Oishi T, Uezumi A, Kanaji A, Yamamoto N, Yamaguchi A, Yamada H, Tsuchida K (2013). Osteogenic differentiation capacity of human skeletal muscle-derived progenitor cells. PLoS One.

[17] Cho HJ, Cho HJ, Lee HJ (2013). Vascular calcifying progenitor cells possess bidirectional differentiation potentials. PLoS Biol.

[18] Dominici M, Le Blanc K, Mueller I (2006). Minimal criteria for defining multipotent mesenchymal stromal cells. The International Society for Cellular Therapy position statement. Cytotherapy.

[19] Uezumi A, Fukada S, Yamamoto N (2014). Identification and characterization of PDGFRα+ mesenchymal progenitors in human skeletal muscle. Cell Death Dis.

[20] Xu J, Wang Y, Li Z (2022). PDGFRα reporter activity identifies periosteal progenitor cells critical for bone formation and fracture repair. Bone Res.

[21] Alvarez R, Lee HL, Wang CY, Hong C (2015). Characterization of the osteogenic potential of mesenchymal stem cells from human periodontal ligament based on cell surface markers. Int J Oral Sci.

[22] Agarwal S, Loder S, Cholok D (2017). Surgical excision of heterotopic ossification leads to re-emergence of mesenchymal stem cell populations responsible for recurrence. Stem Cells Transl Med.

[23] Bartoletti G, Dong C, Umar M, He F (2020). Pdgfra regulates multipotent cell differentiation towards chondrocytes via inhibiting Wnt9a/beta-catenin pathway during chondrocranial cartilage development. Dev Biol.

[24] Mueller AA, van Velthoven CT, Fukumoto KD, Cheung TH, Rando TA (2016). Intronic polyadenylation of PDGFRα in resident stem cells attenuates muscle fibrosis. Nature.

[25] Harvey T, Flamenco S, Fan CM (2019). A Tppp3(+)Pdgfra(+) tendon stem cell population contributes to regeneration and reveals a shared role for PDGF signalling in regeneration and fibrosis. Nat Cell Biol.

